# A strontium isoscape for the Conchucos region of highland Peru and its application to Andean archaeology

**DOI:** 10.1371/journal.pone.0248209

**Published:** 2021-03-30

**Authors:** Eden Washburn, Jason Nesbitt, Bebel Ibarra, Lars Fehren-Schmitz, Vicky M. Oelze

**Affiliations:** 1 Department of Anthropology, University of California Santa Cruz, Santa Cruz, California, United States of America; 2 Department of Anthropology, Tulane University, New Orleans, Louisiana, United States of America; University of Florence, ITALY

## Abstract

Strontium isotope (^87^Sr/^86^Sr) analysis of human skeletal remains is an important method in archaeology to examine past human mobility and landscape use. ^87^Sr/^86^Sr signatures of a given location are largely determined by the underlying bedrock, and these geology specific isotope signatures are incorporated into skeletal tissue through food and water, often permitting the differentiation of local and non-local individuals in past human populations. This study presents the results of a systematic survey of modern flora and fauna (n = 100) from 14 locations to map the bioavailable ^87^Sr/^86^Sr signatures of the Conchucos region, an area where the extent of geologic variability was previously unknown. We illustrate the necessity to examine the variation in ^87^Sr/^86^Sr values of the different geological formations available to human land use to document the range of possible local ^87^Sr/^86^Sr values. Within the Conchucos region we found significant variation in environmental ^87^Sr/^86^Sr values (0.7078–0.7214). The resulting isoscape represents the largest regionally specific bioavailable ^87^Sr/^86^Sr map (3,840 km^2^) to date for the Andes, and will serve as a baseline for future archaeological studies of human mobility in this part of the Peruvian highlands.

## Introduction

The study of mobility and migration are important topics in contemporary archaeology [1]. While human mobility can be studied using a variety of archaeological indicators, recent years have witnessed a marked increase in investigations that employ isotopic analyses of human remains to study ancient population movements [2–11]. Molecular methods enable researchers to focus primarily on the individual [12–16], and can elucidate aspects of human behavior such as mobility and landscape utilization that are otherwise difficult to observe [15]. Because strontium has the unique ability to substitute for calcium (Ca) in the hydroxyapatite of bone and tooth enamel, the use of strontium isotopes (^87^Sr/^86^Sr) analysis in skeletal remains can provide insight into past human and other animal movement throughout a landscape. When locally available nutrients are consumed, ^87^Sr/^86^Sr values in an organism reflect the bioavailable (i.e., only the strontium which makes its way into the food chain; see further discussion below) signature of the immediate geological location in which an individual lived. Nevertheless, the use of ^87^Sr/^86^Sr isotope analysis to identify non-local individuals and their potential place of origin, relies on an accurate characterization of local ^87^Sr/^86^Sr ranges, either through statistical spatial modeling or by testing modern/archaeological proxy materials to establish local baselines.

In the Andes, ^87^Sr/^86^Sr isotope analysis has been used to address a wide range of fundamentally important questions surrounding human life and interaction [17–52]. Significant variation in the geology of the Andes makes the use of strontium isotopic analysis a useful tool in determining “local” vs “non-local” inhabitants of an archaeological site [15, 45, 53]. The Andes mountains are composed of many folded geological layers [54–56] that generally run in parallel from north to south and these geological formations can be relatively narrow and stacked close together. As a result, inhabitants of a specific archaeological site may have encountered (or frequented) multiple geological formations, thus making the identification of potential migrants more challenging. For example, the physical location of an archaeological site may not have been located in the same geological formation in which food was cultivated or herding and hunting was conducted.

**Fig 1:**
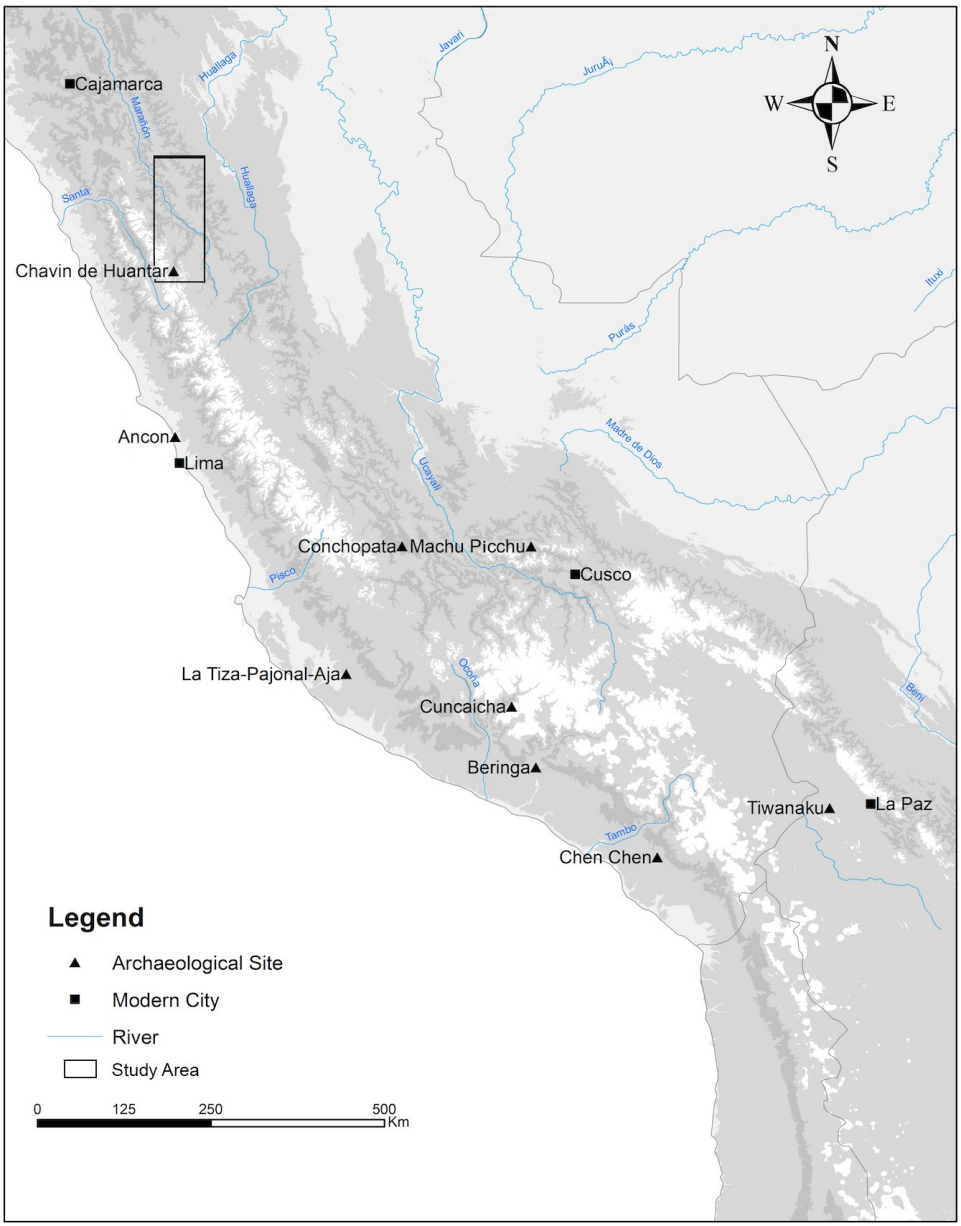
Map of Peru showing the Conchucos region, Department of Ancash (identified by black rectangle) as well as the locations of previous ^87^Sr/^86^Sr isotope studies throughout Peru. Map was produced in ArcGIS 10.4, with all subsequent layout and design preformed in Photoshop CC 14.2.

In regions where the extent of geologic variation and the range of ^87^Sr/^86^Sr values are known, ^87^Sr/^86^Sr analysis can be used to track mobility and illuminate processes of interaction. In Peru, the majority of ^87^Sr/^86^Sr isotope studies have been situated along the Pacific coast, in the southern Andes and/or west of the Cordillera Blanca. The limited number of ^87^Sr/^86^Sr studies within the highland valley systems of the north central Andes have resulted in an underestimation of the geologic complexity throughout the region. Here we add to the growing body of ^87^Sr/^86^Sr studies in the Andes, and present the first regional map of the variation in bioavailable ^87^Sr/^86^Sr values of the Conchucos region of highland Ancash (Fig 1), a region with a rich archaeological history [57]. Our study also raises questions related to what may constitute isotope-based determinations of local and non-local populations in Andean archaeology.

## Background

### Strontium geochemistry

The trace element strontium (Sr) is found in extremely low concentrations in bedrock, groundwater, soil, plants, and animals. Sr is composed of different percentages of the following four stable isotopes: ^84^Sr (~0.56%), ^86^Sr (~9.87%), ^87^Sr (~7.04%) and ^88^Sr (~82.53%) [74, 75]. Of these four isotopes, ^87^Sr is radiogenic and formed over time by the radioactive decay of rubidium (^87^Rb) in the bedrock, which has a half-life of ~4.88 x 10^10^ years. As a result, specific concentrations of ^87^Sr in the environment are a result of a bedrock’s age and Rb content [58–60]. Sr enters the biosphere through uptake from the substrate by plants and cycles through food webs, into for example, the tissues of both animals and humans.

However, not all Sr in bedrock is uniformly weathered into the soil and water [15, 61, 62]. Various minerals found within a single bedrock unit can have considerable variability in their ^87^Sr/^86^Sr values. For example, granite can have two feldspars with radically differing ^87^Sr/^86^Sr values (plagioclase and potassium feldspars) depending on which section is measured [15]. As such, biologically available ^87^Sr/^86^Sr, which is soluble and is taken up by biotic agents, can substantially differ in its values between the lithosphere and the biosphere [12, 15, 63, 64]. As a result, direct bedrock ^87^Sr/^86^Sr measurements typically conducted for geological dating studies [65–70] are not necessarily accurate for applications in archaeological science. Besides Sr deriving from the weathering of local bedrock, atmospheric and surface sources, such as rainfall, rivers, sea-spray, and wind-dust, also contribute to the bioavailable Sr in the food chain [15, 59, 71–74]. Modern anthropogenic Sr contaminations can be introduced through industrial fertilizers and even via dust from large scale construction sites [15, 59, 71, 75].

As organisms consume locally available food and water, these sources of Sr are mixed and incorporated into the organism’s tissue [12, 42, 63]. In contrast to many commonly utilized light isotope systems, the isotopic composition of Sr does not change or fractionate during biological processes [63]. This is because the mass differences between the four Sr isotopes are relatively small [13, 62, 63, 76]. As a result, the ^87^Sr/^86^Sr values measured in flora and fauna vary mainly based on the age of the bedrock on which they sourced their nutrients. Very old bedrock with high Rb/Sr ratios will have the highest ^87^Sr/^86^Sr values today [58, 77]. Examples of geological deposits that have relatively high Rb/Sr ratios include clay-rich rocks such as shale, or igneous rocks that have high silica content, such as granite, with ^87^Sr/^86^Sr values up to 0.715 [13]. In contrast, geologically young rocks and sediments will have low Rb/Sr ratios and typically have ^87^Sr/^86^Sr less than 0.706 [e.g., 78]. To infer the biologically available ^87^Sr/^86^Sr values in an area, recent studies commonly use samples of uncontaminated environmental sources of local origin such as plants and small animals [59, 75, 79, 80], water [72, 81, 82] and soil [59] samples.

### ^87^Sr/^86^Sr analysis in archaeology

While traditional archaeological approaches primarily rely on artifactual indications of population movement [16], the use of ^87^Sr/^86^Sr data obtained from human skeletal material allows researchers to directly examine individual mobility. Most studies employing ^87^Sr/^86^Sr analysis focus on the study of human skeletal remains from archaeological sites with the intention of identifying immigrants and to track residential mobility in the past [e.g., 3, 7–9, 83, 84]. ^87^Sr/^86^Sr studies have also been applied to address broader sociocultural questions relating to imperial strategies [17, 27, 28, 31, 32, 43, 51, 84, 85], colonization [31, 85–88], post-marital residential patterns [89–92], identity [37, 51, 93–95] and warfare [22, 36, 38, 96, 97].

As ^87^Sr/^86^Sr isotope analysis has been applied to address a wide range of archaeologically significant questions, methods for determining local ^87^Sr/^86^Sr ranges in the environment also continue to improve. Originally, researchers determined the local range of ^87^Sr/^86^Sr values as a two-standard deviation (±2σ) range around the average ^87^Sr/^86^Sr value measured in all archaeological samples from a site, characterizing outliers as non-local individuals [15, 16]. This tends to produce a conservative estimate of non-locals in a population and may inadvertently underestimate the number of non-locals in a sample [15, 42, 98].

Given the potential challenges with defining local ranges based on mean calculations of the ^87^Sr/^86^Sr values of an ancient (and potentially highly mobile) human population, researchers now commonly sample local (both archaeological and modern) fauna and flora as proxies for locally bioavailable ^87^Sr/^86^Sr [5, 15–16, 20, 22, 32, 44, 98–101]. There are however, several considerations that should be made in sample selection [16]. In archaeological fauna, it is often unclear if animals were kept locally, remotely, or if they were subject to exchange. Depending on the source, modern domestic fauna may not reflect local ^87^Sr/^86^Sr values if they were fed imported, non-local foods, and/or if fodder was exposed to exogenic Sr though industrial fertilizers [15, 16, 59, 62]. Animals, or animal products, purchased from local markets where their geographic origin and/or the origin of their fodder may be unclear, can make associating the obtained ^87^Sr/^86^Sr data to a specific geological formation with the necessary certainty difficult [15, 31, 32].

In recent years there has also been an increased effort to create large-scale isoscapes, a spatially explicit prediction of isotopic variation across landscapes [42, 102–105]. An isoscape considers all published ^87^Sr/^86^Sr data for a given region and uses this dataset to extrapolate the extent of possible ^87^Sr/^86^Sr values across large geographic areas [71, 75, 106–111]. While these studies provide invaluable insight into the nature of past mobility on a population-wide pan-regional scale, they are dependent on the amount and quality of data used to generate the isoscape.

There are many approaches to conducting ^87^Sr/^86^Sr isotope research and each of these methods have advantages and limitations depending on the research questions and resolution of the data. In this study we present a detailed regional mapping project that emphasizes the collection of environmental samples of biologically available ^87^Sr/^86^Sr, both within archaeological sites, as well as from the surrounding geological formations. This regional isoscape can then be applied to the study of human and animal mobility within the region.

### Geology of the Peruvian Andes

The Central Andes are divided into the Cordillera Occidental to the west and the Cordillera Oriental to the east. The Cordillera Occidental is largely composed of late Cenozoic volcanic rocks such as andesites and Mesozoic formations. Age of the Cenozoic volcanic rock increases from the northern Andes to the southern Andes, and as a result the ^87^Sr/^86^Sr values are generally higher in the southern part of the Andes [78, 112]. ^87^Sr/^86^Sr values reported from late Cenozoic volcanic rocks in Ecuador exhibit ^87^Sr/^86^Sr values of 0.70431±0.00016 (1σ, n = 23) [112], while exposed bedrock samples from similar geologic formations in northern Chile exhibit mean ^87^Sr/^86^Sr values of 0.70646 ±0.00020 (1σ, n = 8) [78]. The Cordillera Oriental in the east is mainly comprised of Paleozoic geology. These formations generally have higher ^87^Sr/^86^Sr values than the western Cordillera; however, their ^87^Sr/^86^Sr values have not yet been measured in bedrock [61, 113]. In addition, on a broad pan-regional scale, ^87^Sr/^86^Sr values seemingly increase along a west to east gradient, with lower values along the Pacific coast (i.e., ~0.7038) and generally higher values moving inland to the east (~0.7239) [42].

**Fig 2:**
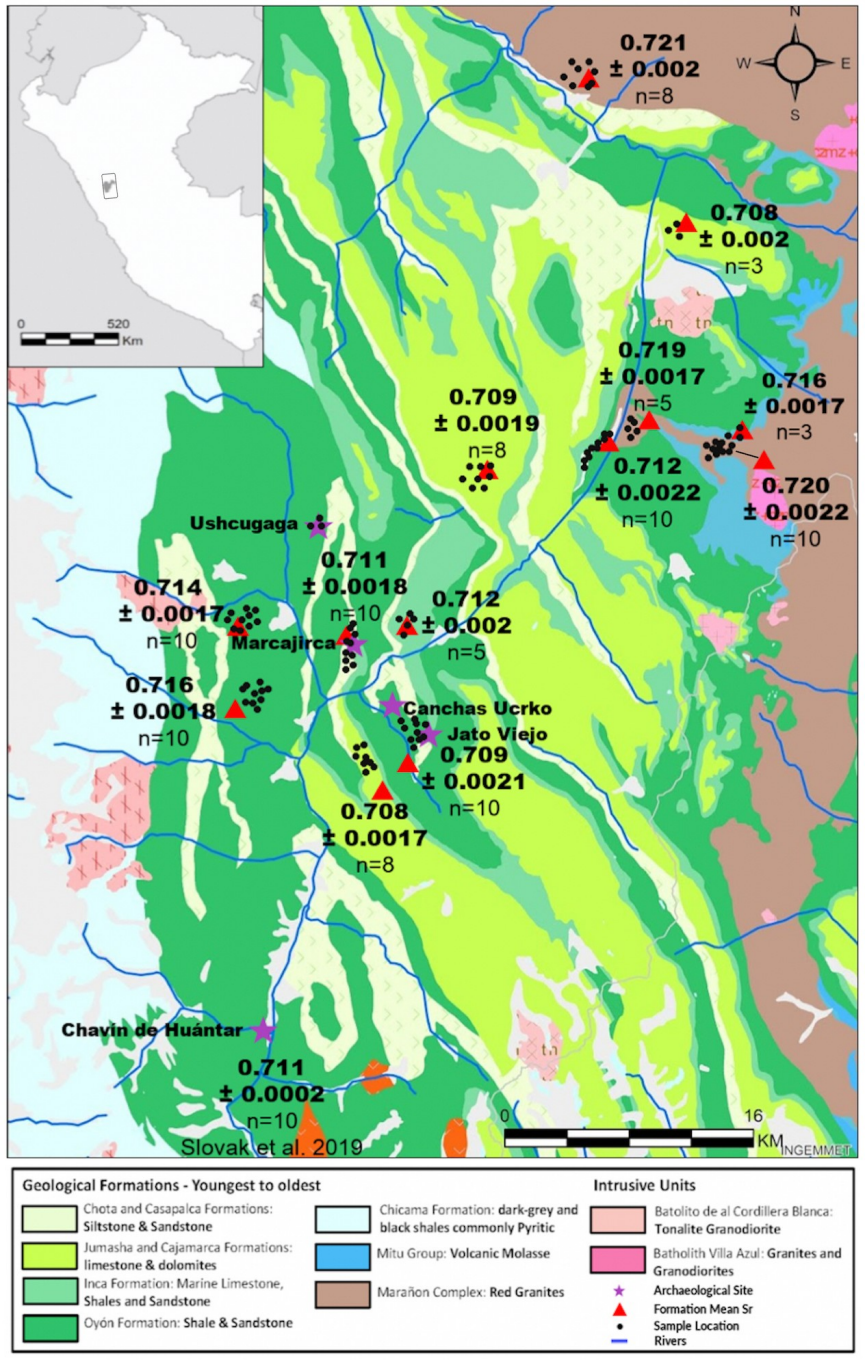
^87^Sr/^86^Sr isoscape of the Conchucos region in the north-central Peruvian highlands. Environmental reference samples include grass (*Stipa ichu)* and snail shells (Bulimulidae). Mean ^87^Sr/^86^Sr values were calculated for each geological formation and are presented along with mean standard error. Individual sampling locations are identified by black dots. Map was produced in ArcGIS 10.4, with all subsequent layout and design preformed in Photoshop CC 14.2.

Geographical and isotopic descriptions of the Andes in these broad terms do not adequately capture the geological complexity of this region, as depicted in Fig 2. It is because of this geological diversity that archaeologists are employing molecular tools such as ^87^Sr/^86^Sr analysis to address questions surrounding human life histories and population movements within the challenging landscape of the Andes [e.g., 17, 20–22, 50, 100].

## Material and study region

The study area consists of a broad swath of the eastern highlands of north-central Peru known as the Conchucos region. Conchucos is an intermontane valley system situated on the southeastern side of the Cordillera Blanca and is characterized by several rivers that drain into the Marañón River, one of the major tributaries of the Amazon. Our study focuses on sample collection over an area of 2,640 km^2^ that includes the Huaritambo, Mosna/Puccha, and Marañón rivers. This region is archaeologically rich [e.g., 70], with archaeological sites dating from ca. 1100 BCE until the 16^th^ century [e.g., 114–126].

As illustrated in Fig 2, the Conchucos region is geologically diverse. The predominant geology comprises folded Mesozoic sedimentary rock formations, including sandstones, dark shales, and carbonates (limestone, marls, and dolomites), as well as metamorphic rocks like quartzite and slate [127–129]. The entire region is shaped by these folded and uplifted layers of bedrock that causes the repetition of specific geologic units over a broad area. This is important to consider when defining the categories of local and non-local populations in the archaeological record based on ^87^Sr/^86^Sr values, as similar geological units can be found throughout the landscape. Towards the north and east the study area is bordered by the geological Marañón Group. Dating to the Proterozoic, Marañón Group rocks are much older than the other formations and consist of meta-sedimentary schists, gneiss, and red sandstone [130].

To assess bioavailable ^87^Sr/^86^Sr values, we collected empty shells of modern terrestrial snails (Bulimulidae), as well as wild perennial grasses abundant in the Peruvian highlands (i.e. *Stipa ichu*) (Table 1; Fig 2). Snail shells are plentiful on the landscape and make it unnecessary to obtain live animals. Snails are additionally limited in the extent of their movement throughout their lifetime and can therefore be considered representative of local variability in bioavailable ^87^Sr/^86^Sr [83, 131, 132]. Sr is deposited in the snail shell, where it substitutes for its main component Ca [133]. Plant ^87^Sr/^86^Sr values reflect the ^87^Sr/^86^Sr values in the immediate local soil, as well as ^87^Sr/^86^Sr admixture introduced by rainwater and atmospheric dust [131].

**Table 1:** Results of ^87^Sr/^86^Sr environmental sampling from six geological formations within the Conchucos region of Peru; sorted by sampling location.

Lab Code	Sample	Geological Formation	Geological Age	Latitude	Longitude	^87^Sr/^86^Sr	StdErr (%)
HAGS 1	Bulimulidae	Casapalca, Chota, Huaylas	Cenozoic to Mesozoic	S 09°22’23.8"	W077°07’49.5"	0.7113	0.0015
HAGS 2	Bulimulidae	Casapalca, Chota, Huaylas	Cenozoic to Mesozoic	S 09°22’11.91"	W077°07’49.39"	0.7111	0.00147
HAGS 3	Bulimulidae	Casapalca, Chota, Huaylas	Cenozoic to Mesozoic	S 09°22’14.09"	W077°07’49.47"	0.7104	0.00232
HAGS 4	Bulimulidae	Casapalca, Chota, Huaylas	Cenozoic to Mesozoic	S 09°22’15.74"	W077°07’49.56	0.71140	0.0022
HAGS 5	Bulimulidae	Casapalca, Chota, Huaylas	Cenozoic to Mesozoic	S 09°22’17.40"	W077°07’49.71"	0.7114	0.00159
HAGS 6	*Stipa ichu*	Casapalca, Chota, Huaylas	Cenozoic to Mesozoic	S 09°22’27.7"	W077°07’50.3"	0.7108	0.00247
HAGS 7	*Stipa ichu*	Casapalca, Chota, Huaylas	Cenozoic to Mesozoic	S 09°22.24.9"	W077°07’49.2"	0.7107	0.0018
HAGS 8	*Stipa ichu*	Casapalca, Chota, Huaylas	Cenozoic to Mesozoic	S 09°22’18.33"	W077°07’49.76"	0.7109	0.00145
HAGS 9	*Stipa ichu*	Casapalca, Chota, Huaylas	Cenozoic to Mesozoic	S 09°22’19.66"	W077°07’49.11"	0.7113	0.00185
HAGS 10	*Stipa ichu*	Casapalca, Chota, Huaylas	Cenozoic to Mesozoic	S 09°22’21.26"	W077°07’49.93"	0.7109	0.00154
**Mean**						**0.71102**	**0.0018**
HAGS 11	Bulimulidae	Casapalca, Chota, Huaylas	Cenozoic to Mesozoic	S 09°13’56.7"	W076°58’18.7"	0.7113	0.00225
HAGS 12	Bulimulidae	Casapalca, Chota, Huaylas	Cenozoic to Mesozoic	S 09°13’57.3"	W076°58’18.8"	0.7108	0.00243
HAGS 13	Bulimulidae	Casapalca, Chota, Huaylas	Cenozoic to Mesozoic	S 09°13’48.0"	W076°58’14.2"	0.7139	0.00281
HAGS 14	Bulimulidae	Casapalca, Chota, Huaylas	Cenozoic to Mesozoic	S 09°13’55.5"	W076°58’19.3"	0.7102	0.00285
HAGS 15	Bulimulidae	Casapalca, Chota, Huaylas	Cenozoic to Mesozoic	S 09°13’57.3"	W076°58’17.2"	0.7123	0.0029
HAGS 16	*Stipa ichu*	Casapalca, Chota, Huaylas	Cenozoic to Mesozoic	S 09°13’47.2"	W076°58’13.8"	0.7141	0.00166
HAGS 17	*Stipa ichu*	Casapalca, Chota, Huaylas	Cenozoic to Mesozoic	S 09°13’55.70"	W076°58’17.73"	0.7107	0.00145
HAGS 18	*Stipa ichu*	Casapalca, Chota, Huaylas	Cenozoic to Mesozoic	S 09°13’50.43"	W076°58’14.17"	0.7138	0.00206
HAGS 19	*Stipa ichu*	Casapalca, Chota, Huaylas	Cenozoic to Mesozoic	S 09°13’47.2"	W076°58’13.8"	0.7113	0.00162
HAGS 20	*Stipa ichu*	Casapalca, Chota, Huaylas	Cenozoic to Mesozoic	S 09°14’25.2"	W076°54’16.3"	0.7109	0.00207
**Mean**						**0.7119**	**0.00222**
HAGS 21	Bulimulidae	Jumasha, Celendin, and Cajamarca	Mesozoic	S 09°23’34.7"	W077°08’09.9"	0.7083	0.00182
HAGS 22	Bulimulidae	Jumasha, Celendin, and Cajamarca	Mesozoic	S 09°23’30.3"	W077°08’06.6"	0.7081	0.00143
HAGS 23	Bulimulidae	Jumasha, Celendin, and Cajamarca	Mesozoic	S 09°23’35.03"	W077°08’08.62"	0.7078	0.00167
HAGS 24	Bulimulidae	Jumasha, Celendin, and Cajamarca	Mesozoic	S 09°23’35.77"	W077°08’10.13"	0.7089	0.00175
HAGS 25	Bulimulidae	Jumasha, Celendin, and Cajamarca	Mesozoic	S 09°23’34.77"	W077°08’11.45"	0.7084	0.00146
HAGS 26	*Stipa ichu*	Jumasha, Celendin, and Cajamarca	Mesozoic	S 09°23’30.3"	W077°08’06.6"	0.7078	0.00232
HAGS 27	*Stipa ichu*	Jumasha, Celendin, and Cajamarca	Mesozoic	S 09°23’32.97"	W077°08’11.14"	0.7081	0.00171
HAGS 28	*Stipa ichu*	Jumasha, Celendin, and Cajamarca	Mesozoic	S 09°23’31.99"	W077°08’06.09"	0.7087	0.00166
**Mean**						**0.7083**	**0.0017**
HAGS 29	Bulimulidae	Jumasha, Celendin, and Cajamarca	Mesozoic	S 09°15’7.98"	W077°03’22.52"	0.7083	0.00175
HAGS 30	Bulimulidae	Jumasha, Celendin, and Cajamarca	Mesozoic	S 09°15’3.73"	W077°02’54.14"	0.7092	0.00156
HAGS 31	Bulimulidae	Jumasha, Celendin, and Cajamarca	Mesozoic	S 09°15’27.39""	W077°03’ 37.08"	0.7079	0.00167
HAGS 32	Bulimulidae	Jumasha, Celendin, and Cajamarca	Mesozoic	S 09°15’39.97"	W077°03’53.99"	0.7087	0.0019
HAGS 33	Bulimulidae	Jumasha, Celendin, and Cajamarca	Mesozoic	S 09°16’16.30"	W077°03’14.55"	0.7094	0.00146
HAGS 34	*Stipa ichu*	Jumasha, Celendin, and Cajamarca	Mesozoic	S 09°16’5.40"	W077°02’52.40"	0.7093	0.00232
HAGS 35	*Stipa ichu*	Jumasha, Celendin, and Cajamarca	Mesozoic	S 09°14’ 33.43	W077°03’22.03"	0.7084	0.00275
HAGS 36	*Stipa ichu*	Jumasha, Celendin, and Cajamarca	Mesozoic	S 09°14’38.64"	W077°04’30.78"	0.7087	0.00163
**Mean**						**0.7087**	**0.0019**
HAGS 37	Bulimulidae	Jumasha, Celendin, and Cajamarca	Mesozoic	S 09°6’12.97"	W076°56’54.33"	0.7082	0.0024
HAGS 38	*Stipa ichu*	Jumasha, Celendin, and Cajamarca	Mesozoic	S 09°6’24.25"	W076°56’31.54"	0.7081	0.002
HAGS 39	*Stipa ichu*	Jumasha, Celendin, and Cajamarca	Mesozoic	S 09°6’14.00"	W076°56’35.72"	0.7087	0.0018
**Mean**						**0.7083**	**0.002**
HAGS 40	Bulimulidae	Oyón, Huaalhuani and Murco	Mesozoic	S 09°24’24.3"	W077°06’09.7"	0.7092	0.00154
HAGS 41	Bulimulidae	Oyón, Huaalhuani and Murco	Mesozoic	S 09°24’23.06"	W077°06’09.62"	0.7091	0.00208
HAGS 42	Bulimulidae	Oyón, Huaalhuani and Murco	Mesozoic	S 09°24’23.89"	W077°06’09.40"	0.7087	0.00181
HAGS 43	Bulimulidae	Oyón, Huaalhuani and Murco	Mesozoic	S 09°24’22.00"	W077°06’09.94"	0.7092	0.00193
HAGS 44	Bulimulidae	Oyón, Huaalhuani and Murco	Mesozoic	S 09°24’24.3"	W077°06’09.72"	0.7108	0.00174
HAGS 45	*Stipa ichu*	Oyón, Huaalhuani and Murco	Mesozoic	S 09°24’22.12"	W077°06’10.46"	0.7092	0.00283
HAGS 46	*Stipa ichu*	Oyón, Huaalhuani and Murco	Mesozoic	S 09°24’23.51"	W077°06’10.07"	0.7089	0.00293
HAGS 47	*Stipa ichu*	Oyón, Huaalhuani and Murco	Mesozoic	S 09°24’24.88"	W077°06’09.53"	0.7084	0.00167
HAGS 48	*Stipa ichu*	Oyón, Huaalhuani and Murco	Mesozoic	S 09°24’24.88"	W077°06’09.10"	0.7082	0.00256
HAGS 49	*Stipa ichu*	Oyón, Huaalhuani and Murco	Mesozoic	S 09°24’24.34"	W077°06’09.25"	0.7089	0.00184
**Mean**						**0.7091**	**0.0021**
HAGS 50	Bulimulidae	Oyón, Huaalhuani and Murco	Mesozoic	S 09°23’21.9"	W077°10’31.9"	0.7158	0.0016
HAGS 51	Bulimulidae	Oyón, Huaalhuani and Murco	Mesozoic	S 09°23’23.5"	W077°10’34.0"	0.7179	0.00187
HAGS 52	Bulimulidae	Oyón, Huaalhuani and Murco	Mesozoic	S 09°23’22.8"	W077°10’34.6"	0.7172	0.00131
HAGS 53	Bulimulidae	Oyón, Huaalhuani and Murco	Mesozoic	S 09°23’21.23"	W077°10’33.32"	0.7164	0.00204
HAGS 54	Bulimulidae	Oyón, Huaalhuani and Murco	Mesozoic	S 09°23’21.68"	W077°10’35.90"	0.7159	0.00184
HAGS 55	*Stipa ichu*	Oyón, Huaalhuani and Murco	Mesozoic	S 09°23’21.2"	W077°10’31.7"	0.7164	0.00251
HAGS 56	*Stipa ichu*	Oyón, Huaalhuani and Murco	Mesozoic	S 09°23’20.2"	W077°10’32.9"	0.7151	0.00158
HAGS 57	*Stipa ichu*	Oyón, Huaalhuani and Murco	Mesozoic	S 09°23.329"	W077°10.33.7"	0.7157	0.00224
HAGS 58	*Stipa ichu*	Oyón, Huaalhuani and Murco	Mesozoic	S 09°23’22.2"	W077°10’32.8"	0.7159	0.00175
HAGS 59	*Stipa ichu*	Oyón, Huaalhuani and Murco	Mesozoic	S 09°23’22.2"	W077°10’33.2"	0.7161	0.00145
**Mean**						**0.7162**	**0.0018**
HAGS 60	Bulimulidae	Oyón, Huaalhuani and Murco	Mesozoic	S 09°2106.9"	W077°11’23.0"	0.7142	0.0016
HAGS 61	Bulimulidae	Oyón, Huaalhuani and Murco	Mesozoic	S 09°21’08.2"	W077°11’20.2"	0.7133	0.00242
HAGS 62	Bulimulidae	Oyón, Huaalhuani and Murco	Mesozoic	S 09°21’05.4"	W077°11’15.9"	0.7139	0.00163
HAGS 63	Bulimulidae	Oyón, Huaalhuani and Murco	Mesozoic	S 09°21’05.8"	W077°11’14.7"	0.7141	0.0007
HAGS 64	Bulimulidae	Oyón, Huaalhuani and Murco	Mesozoic	S 09°21’07.27"	W077°11’17.47"	0.7128	0.00236
HAGS 65	*Stipa ichu*	Oyón, Huaalhuani and Murco	Mesozoic	S 09°21’05.2"	W077°11’18.5"	0.7134	0.00197
HAGS 66	*Stipa ichu*	Oyón, Huaalhuani and Murco	Mesozoic	S 09°21’07.1"	W077°11’12.6"	0.7142	0.00307
HAGS 67	*Stipa ichu*	Oyón, Huaalhuani and Murco	Mesozoic	S 09°21’07.37"	W077°11’15.74"	0.7138	0.00175
HAGS 68	*Stipa ichu*	Oyón, Huaalhuani and Murco	Mesozoic	S 09°21’03.28"	W077°11’15.24"	0.7141	0.0006
HAGS 69	*Stipa ichu*	Oyón, Huaalhuani and Murco	Mesozoic	S 09°21’03.10"	W077°11’20.21"	0.7134	0.00247
**Mean**						**0.7137**	**0.0019**
HAGS 70	Bulimulidae	Inca, Pariahuanca, chúlec Pariatambo	Mesozoic	S 09°21’2.44 "	W077°5’35.66"	0.7123	0.00134
HAGS 71	Bulimulidae	Inca, Pariahuanca, chúlec Pariatambo	Mesozoic	S 09°21’39.05"	W077°5’31.39"	0.7121	0.00252
HAGS 72	*Stipa ichu*	Inca, Pariahuanca, chúlec Pariatambo	Mesozoic	S 09°21’23.49"	W077°5’14.96"	0.7129	0.00162
HAGS 73	*Stipa ichu*	Inca, Pariahuanca, chúlec Pariatambo	Mesozoic	S 09°21’7.91"	W077°5’59.54"	0.7121	0.00153
HAGS 74	*stipa ichu*	Inca, Pariahuanca, chúlec Pariatambo	Mesozoic	S 09°21’42.17"	W077°5’40.25"	0.7126	0.00234
**Mean**						**0.7124**	**0.002**
HAGS 75	*Stipa ichu*	Pucará Group	Paleozoic	S 09°14’10.39 "	W076°54’43.81"	0.7163	0.00231
HAGS 76	*Stipa ichu*	Pucará Group	Paleozoic	S 09°14’15.54 "	W076°55’1.53"	0.7154	0.00123
HAGS 77	*Stipa ichu*	Pucará Group	Paleozoic	S 09°14’5.98"	W076°55’15.38"	0.7158	0.00166
**Mean**						**0.7158**	**0.0017**
HAGS 78	Bulimulidae	Marañón Group	Neoproterozoic	S 09°14’25.92"	W076°54’17.51"	0.7198	0.00179
HAGS 79	Bulimulidae	Marañón Group	Neoproterozoic	S 09°14’28.26"	W076°54’18.67"	0.7207	0.00212
HAGS 80	Bulimulidae	Marañón Group	Neoproterozoic	S 09°14’29.93"	W076°54’16.72"	0.7212	0.00146
HAGS 81	Bulimulidae	Marañón Group	Neoproterozoic	S 09°14’28.10"	W076°54’14.52"	0.7214	0.00237
HAGS 82	Bulimulidae	Marañón Group	Neoproterozoic	S 09°14’30.79"	W076°54’12.78"	0.7202	0.00253
HAGS 83	*Stipa ichu*	Marañón Group	Neoproterozoic	S 09°14’33.0"	W076°54’09.6"	0.7159	0.00223
HAGS 84	*Stipa ichu*	Marañón Group	Neoproterozoic	S 09°14’29.7"	W076°54’14.9"	0.7197	0.00358
HAGS 85	*Stipa ichu*	Marañón Group	Neoproterozoic	S 09°14’29.4"	W076°54’18.2"	0.7215	0.00253
HAGS 86	*Stipa ichu*	Marañón Group	Neoproterozoic	S 09°14’29.1"	W076°54’19.6"	0.7187	0.00243
HAGS 87	*Stipa ichu*	Marañón Group	Neoproterozoic	S 09°14’32.20"	W076°54’10.85"	0.7208	0.00126
**Mean**						**0.7200**	**0.0022**
HAGS 88	Bulimulidae	Marañón Group	Neoproterozoic	S 09°14’ 8.26"	W076°57’45.70"	0.7179	0.00172
HAGS 89	Bulimulidae	Marañón Group	Neoproterozoic	S 09°13’56.75"	W076°57’43.34"	0.7187	0.00125
HAGS 90	*Stipa ichu*	Marañón Group	Neoproterozoic	S 09°14’ 5.09"	W076°57’59.32"	0.7192	0.00223
HAGS 91	*Stipa ichu*	Marañón Group	Neoproterozoic	S 09°13’54.91 "	W076°58’3.08"	0.7194	0.00164
HAGS 92	*Stipa ichu*	Marañón Group	Neoproterozoic	S 09°14’ 12.27"	W076°58’1.29"	0.7184	0.00143
**Mean**						**0.7187**	**0.0017**
HAGS 93	Bulimulidae	Marañón Group	Neoproterozoic	S 09°0’6.11"	W077°1’2.29"	0.7208	0.00223
HAGS 94	Bulimulidae	Marañón Group	Neoproterozoic	S 09°0’5.07 "	W077°1’32.08"	0.7206	0.00235
HAGS 95	Bulimulidae	Marañón Group	Neoproterozoic	S 09°0’46.10 "	W077°0’59.93"	0.7203	0.00124
HAGS 96	Bulimulidae	Marañón Group	Neoproterozoic	S 09°0’22.11"	W077°0’45.79."	0.7212	0.00246
HAGS 97	Bulimulidae	Marañón Group	Neoproterozoic	S 09°0’13.72"	W077°0’29.21"	0.7205	0.00129
HAGS 98	*Stipa ichu*	Marañón Group	Neoproterozoic	S 09°0’3.83"	W077°1’22.60"	0.7206	0.00238
HAGS 99	*Stipa ichu*	Marañón Group	Neoproterozoic	S 09°0’37.89 "	W077°0’54.38"	0.7196	0.00229
HAGS 100	*Stipa ichu*	Marañón Group	Neoproterozoic	S 09°0’37.57 "	W077°0’54.78"	0.7198	0.00231
**Mean**						**0.7205**	**0.020**

During field sampling, major geological formations in the region were identified using a geological map [129]. We obtained 100 modern environmental reference samples from 14 sampling sites in six geological units covering a 3,840 km^2^ area of the Conchucos region. In each geological unit, we selected sampling locations where anthropogenic contamination through fertilizers or other pollutants were unlikely, as there were no signs of use through agriculture and there was considerable distance to roads and/or towns. At each location we collected snail shells from the surface alongside several samples of *Stipa ichu* (3–10 plants/unit). Each sample location was recorded via a hand-held GPS. Import permits for all plants and snail shells were granted by the United States Department of Agriculture, Animal and Plant Health Inspection Service (Permit Number: PCIP-18-00364). No export permits were required for the sample material used in this study.

## Methods

Sample preparation was conducted in the Primate Ecology and Molecular Anthropology laboratory (PEMA) at the University of California at Santa Cruz (UCSC). Snail shells were repeatedly rinsed with ddH_2_O in an ultrasonic bath to remove any attached sediment. Snail shells were then broken into smaller fragments, placed in individual beakers with ultrapure acetone, rinsed in an ultrasonic bath for another 15 minutes to remove any potential contaminants on the shell surface and were then set to dry. Plant samples (2g of well- dried plant material) and snail shells (~300mg) were then ashed at 800°C for 12 hours in a muffle furnace. The remaining ash (n = 100 samples) was transferred to the UCSC W.M. Keck Isotope Laboratory clean room, where 20mg of ash from each sample was weighed into clean Teflon beakers and digested for 2 hours in 2ml of 65% HNO_3_ on a hot plate set to 120°C. Due to the cell structure of plant material, complete digestion of plant ash was difficult, thus these samples were subjected to a microwave digestion in an Anton Paar Multiwave GO Microwave Digestion System. Ashed plant material was combined with 8ml of 65% HNO_3_ and 1ml of 6M HCL in a pressure vessel for approximately 30 mins. The dissolved samples of snail shell and microwave digested plants were then placed in open Teflon beakers on a hot plate at 120°C for at least 8 hours to evaporate. Following this, samples were resolved in 1ml of 3M HNO_3_. Each sample was carefully transferred into pre-conditioned chromatography columns containing clean Sr-spec^™^ resin. Samples were reloaded through the resin three times to maximize the amount of Sr attaching to the resin. After 3 washes with 3M HNO_3_, the strontium was eluted from the resin with ultrapure ddH_2_O into clean Teflon beakers and dried down on a hotplate. The remaining sample, again re-dissolved in 5% HNO_3_ was dip checked on the Thermo Finnigan Neptune^™^ MC-ICP-MS instrument to check the concentration of Sr in each sample. Any sample that had a v^88^SR value above 40ppm was diluted down to ~40ppm (v^88^SR). Samples were then measured parallel to the SRM 987 standard, procedural blanks (one/every batch of 9 samples), as well as one clean acid blank after every 5 samples, in a Thermo Finnigan Neptune^™^ MC-ICP-MS.

## Results

We measured ^87^Sr/^86^Sr in 100 environmental samples (50 snail shells and 50 plant samples). Repeated ^87^Sr/^86^Sr measurement of the SRM 987 standard resulted in an average value of 0.7093 ±0.00013. The procedural blanks, one for each batch of nine samples, showed negligible amounts of Sr, indicating no sample cross contamination. ^87^Sr/^86^Sr measured in 50 plant samples range from 0.7071 to 0.7215. ^87^Sr/^86^Sr measured in 50 snail shell samples range from 0.7078 to 0.7214 (Table 1; Fig 2). Mean ^87^Sr/^86^Sr values for each sampling location as well as more detailed information on each geological unit are presented in Table 1.

## Discussion

### The use of ^87^Sr/^86^Sr isoscapes: What does it mean to be a “local”?

Within relatively short distances between sampling locations, we documented considerable differences in mean ^87^Sr/^86^Sr values per geological unit that range from as low as 0.7078 to 0.7212 within 10km distance (Fig 2). This suggests that in these geological settings, ancient farming, animal husbandry and hunting would likely result in the utilization of several larger geological units with distinct geological ages and ^87^Sr/^86^Sr values. We can extend this statement to other locations within the Conchucos region. This finding has important implications for archaeological research interested in understanding past human mobility not only in this specific region, but throughout the Andes.

The analysis of ^87^Sr/^86^Sr values in human skeletal remains is a powerful tool to reconstruct past human mobility. However, the interpretation of ^87^Sr/^86^Sr data are not always straightforward. Individuals with ^87^Sr/^86^Sr values outside the estimated local ^87^Sr/^86^Sr range of a given site are commonly described as having consumed non-local sources of Sr, either by they themselves being non-locals or by consuming non-local foods (i.e. through trade or consuming foods farmed in a geologically distinct region) [1, 7, 8, 30–36, 43, 44]. Whereas those with ^87^Sr/^86^Sr values matching those of the immediate vicinity of the site are considered to have consumed local sources of Sr and were therefore potential residents of that site [1, 7, 8, 30–36, 59]. To address questions surrounding residential mobility requires not only ^87^Sr/^86^Sr data but also nuanced interpretations of archaeological context and potentially the use of light isotopes (i.e. Carbon and Nitrogen) to estimate diet. Our data illustrates that even locally residing individuals can potentially have a range of sources of ^87^Sr/^86^Sr values within a discrete area, depending on where they farmed their plants and produced their animal food. If the ^87^Sr/^86^Sr values measured in enamel are represented in the region surrounding an archaeological site, they may be considered potentially local.

In the highland Andes, archaeological sites are frequently located along ecological boundaries, allowing their inhabitants to exploit multiple ecological zones that cross different geological formations [48, 57, 134–146]. This may have been achieved through connections with populations living in different ecological zones that were linked to each other by exchange and/or kinship relationships, [57, 134, 147–149], or mobile groups who camped at different zones to access resources seasonally [134, 136, 138, 150].

Because the ^87^Sr/^86^Sr value of a given tissue (i.e., bone or tooth enamel) is an average of all the bioavailable ^87^Sr/^86^Sr ingested over the duration of that tissues’ formation [59, 109, 151], the extent of landscape-use related mobility should be considered, especially within regions that are as ecologically and geologically complex as the Andes. If enamel of late forming teeth is used and individuals are frequently consuming dietary items of different geological origin, their ^87^Sr/^86^Sr values will be a mix of the ^87^Sr/^86^Sr values of these consumed food sources. For example, if an adult individual’s ^87^Sr/^86^Sr value does not fit within the bioavailable ^87^Sr/^86^Sr range of a given site that does not necessarily mean this person should be considered non-local. Rather, this may indicate a higher degree of local mobility within the framework of farming and hunting. On the other hand, even though a region is geologically diverse, if individuals were not utilizing the entire landscape human ^87^Sr/^86^Sr values may not be variable. It is for this reason that establishing baseline environmental ^87^Sr/^86^Sr isotope data from within archaeological sites as well as from the surrounding landscape is crucial to a more thorough examination of past human mobility.

For example, in a recent study Slovak and colleagues [44] report the first ^87^Sr/^86^Sr signatures from five human Mariash-Recuay individuals (ca. AD 1–700) buried at the Peruvian highland ceremonial center of Chavín de Huántar (3,180 masl) located in our study region (Fig 2). To establish a local bioavailable ^87^Sr/^86^Sr range, several soil, animal and plant samples collected from within and around the ceremonial center (~2ha) were analyzed [44]. Based on these reference samples, three Chavín human individuals were classified to be of local origin (CdH_38, 39, 40 ^87^Sr/^86^Sr = 0.7111–0.7113), whereas two others with ^87^Sr/^86^Sr values outside the estimated local range (CdH_36 ^87^Sr/^86^Sr = 0.708; CdH_37 ^87^Sr/^86^Sr = 0.706) were considered to be of non-local origin. Slovak and colleagues [44] report potential regions of origin that range from the central coast to the Atacama Desert.

Based on our data, we propose that while it is possible that individual CdH_36 (^87^Sr/^86^Sr value of 0.708) may have migrated to Chavín de Huántar from much further distances, this individual may have had a life history background in the Conchucos region and moved to Chavín de Huántar after early childhood (as premolars and second molars were used in this study). ^87^Sr/^86^Sr values similar to this individual can be found in the vicinity of Chavín de Huántar such as within the Jumasha and Cajamarca formations, only 10 km away, where we report ^87^Sr/^86^Sr values of 0.708 ±0.0017 (Fig 2).

### Comparing a pan-Andean isoscape to our regionally specific ^87^Sr/^86^Sr study

In a recent publication, Scaffidi and Knudson [42] present a pan-Andean isoscape that combines all published ^87^Sr/^86^Sr data prior to 2019 from Peru, and applies geostatistical modeling to generate a predictive model for ^87^Sr/^86^Sr values found within the Andes. As discussed by Scaffidi and Knudson [42], this extensive dataset has the potential to be particularly valuable in regions where baseline environmental sampling is logistically or contextually problematic and/or those regions of Peru lacking ^87^Sr/^86^Sr reference data. Until recently, the majority of ^87^Sr/^86^Sr studies within Peru have taken place either along the Pacific coast or in the southern Andes particularly along the western slopes, with very few studies in the eastern highlands (Fig 2; [e.g., 17–52]). In this isoscape, regions with little to no ^87^Sr/^86^Sr reference data are presented as geologically and isotopically uniform. This affects the projection of ^87^Sr/^86^Sr values for the Conchucos region, for which we present highly variable environmental data.

Scaffidi and Knudson [42] show a general pattern of a west to east gradient of lesser to greater radiogenic values, with lower ^87^Sr/^86^Sr values along the coast (i.e., 0.7038–0.70550 coastal) and generally higher values moving towards the east (0.7177–0.7239). While on a macro-scale and over large distances this distinction is observed, our study suggests that there is also considerable geological variation in the Conchucos region, a small area totaling only 0.4% of Peru. Within the Conchucos region, we document more extensive isotopic variation than initially estimated, including relatively low and relatively radiogenic ^87^Sr/^86^Sr values (0.7078–0.7212).

The geology of the Andes is comprised of closely stacked geological formations that run in parallel from north to south. Our study demonstrates that because each of these geological formations is of distinct geologic age, there are differing ^87^Sr/^86^Sr values represented within close proximity. In contrast to the pan-Andean isoscape created by Scaffidi and Knudson [42], within our localized study region there does not appear to be a west-east trend in ^87^Sr/^86^Sr values. In this region the lowest ^87^Sr/^86^Sr value (0.7078) is found in Jumasha and Cajamarca formations that run in between geological formations with higher ^87^Sr/^86^Sr values (0.7133–0.7178 to the west; 0.7196–0.7208 to the east).

The entire range of documented ^87^Sr/^86^Sr values in all archaeological Andean samples measured to date is 0.7038–0.7234 [42], which is just as broad as reported globally [59]. Interestingly, within the Conchucos isoscape we report a similar range of environmental ^87^Sr/^86^Sr values (0.7078–0.7212). Based on our data, we can predict that this environmental degree of ^87^Sr/^86^Sr variation will be present throughout the Andes. The results of our regional isoscape have the potential to fine tune the resolution of this pan-Andean isoscape.

## Conclusion

This study contributes to the achievements of previous ^87^Sr/^86^Sr isotope studies within Peru by providing a novel and detailed ^87^Sr/^86^Sr isoscape for the previously understudied Conchucos region. We also address the challenge with the application of ^87^Sr/^86^Sr data in making determinations about past human mobility. Our data illustrates the need to consider a larger scope of possibilities to explain why an individual may have an ^87^Sr/^86^Sr value outside of the expected local range.
